# Emergency medicine residency programs: the changing face of graduate medical education

**DOI:** 10.5116/ijme.5a47.8274

**Published:** 2018-01-16

**Authors:** Robyn M. Hoelle, Tami Vega, Zaza Atanelov, Hale Toklu

**Affiliations:** 1 University of Central Florida/HCA GME Consortium North Florida Medical Center, Gainesville, USA

**Keywords:** Emergency medicine residency programs: the changing face of graduate medical education

## To the Editor

Emergency Medicine (EM) is a relatively young speciality, and in just the last few decades has experienced impressive adaptations and radical changes in its graduate medical education (GME) landscape. EM physicians are considered to be forward thinking, flexible and early adopters in education and as clinicians. With rapidly changing graduate medical education needs, educators require new understandings of adult learning that bridges generational differences.^1^ Nationally, the cost of GME had become significant, and without proper and timely intervention would become unsustainable financially, and practically.^2^ EM residency education has been one of the first specialities to evolve and adapt to meet evolving needs of current adult learners and financial sponsors amid controversy.

At the 2007 Academic Emergency Medicine Consensus Conference on knowledge translation in emergency medicine, leading EM researchers and educators concluded that graduating physicians require specific skills to bridge the gap between basic knowledge and clinical practice, specifically, with regard to the principles of evidence-based medicine (EBM).^3^ Significant barriers exist to guiding clinical doctrines and practice with EBM. Identified barriers relate mostly to the teaching and practice variation among faculty members and the paucity of clinically EM relevant high-quality literature.^3^ A Specialty-wide research needs evaluation led to decades of work meeting the needs of our speciality and ultimately our learners and patients.^4^ EM and EBM practices improved through required resident research projects and scholarly activities. This methodology became one of the most effective ways to learn EBM and implement it into practice, as well as, significantly increasing medical literature contributions.^5^ Due to this change and many others, EM scholarly activity became a cornerstone in EM residency.

In 2009, The Accreditation Council for Graduate Medical Education (ACGME) challenged educators to adopt learner-centered^6^ teaching  methodologies to  accommodate the educational needs of this next generation of physician-learners. EM was one of seven specialities who piloted the new Milestone Education Program.^7^ As evidenced by ACGME’s subsequent implementation and positive results of the Outcome Project,^7^ competency-based medical education rapidly gained acceptance in the United States and internationally.^8^ The Outcome Project introduced six general competencies and attempted to create a system that would allow program accreditation based on outcomes. Competency-based medical education became the new standard for all residency programs.^9^^-^^11^ Programs moved away from classic didactic lectures to include more self-directed learning opportunities with experiential learning aids in the development of critical, cognitive and scholarly skills.^1^^,^^8^^,^^12^ In addition, EM has quickly and efficiently implemented the use of social media and online resources, e.g., Twitter, Facebook, blogs, podcasts, live sessions etc. to connect with and meet the needs of the millennial learner.^13^^, ^^14^

In 2015, the Association of American Medical Colleges (AAMC) investigated workforce trends for healthcare providers and reported an estimated physician shortfall between 40,800 and 104,900 physicians by 2030.  Analysis of potential unmet needs by “patient race and ethnicity, region of the United States, and patient residence in metropolitan area” identified specific areas and specialities of need.^15^ In response to financial pressures, political pressures and physician shortages, new medical schools have opened, most of which are set up as for-profit entities.^16^ Specifically, EM physician shortages and barriers for expansion were recognized in the early 2000’s.^17^ The 2008 Hospital-Based Emergency Care: At the Breaking Point report forecasted demand for EM care will continue to increase and resources will be short of projected need.^4^ The addition of new medical school graduates requires the addition of new GME training spots to produce a trained physician workforce. In 2017, Emergency  Medicine  offered 152 more  training  positionsthan any previous year, totalling 2,047 spots. Almost every 99.7%  EM training position open to medical graduates in 2017 was filled in the 2017 Match.^18^ While projected shortfalls still outpace these modest gains in trained physician production, EM educators continue to expand training opportunities.

Amid physician shortages, healthcare access challenges, rising educational costs and skyrocketing healthcare costs, the 2015 Institute of Medicine Report on GME, emphasized the need for a new GME model to increase educational program quality and lower costs to the public in accordance with market consolidation, fiscal pressures, and payers’ new focus on the operating model for academic medicine.^2^ Thus new models suggested investing educational funding and resources into the transformation of community hospitals into training environments. This would expand learning opportunities but divert additional funding from traditional legacy-teaching hospitals.^2^ In recent years, large for-profit hospital corporations have also played an important partin financing and motivating the creation of new residency positions.^16^ Supporting EM residency is a way for large hospital corporations to cultivate their own physicians and have a continuous supply to fill their workforce. Large non-academic management groups have managed EM residencies in the past, but now groups are actively investing millions and starting new programs.^16^^,^^19^

Concerns from EM educators and Florida’s community EM physicians, as discussed in an open town hall forum at the Florida College of Emergency Physicians Symposium By the Sea 2017, include dilution of educator talent, dilution of quality learning experiences (trauma cases, etc.), and  local workforce saturation with program geographic concentration. EM as a speciality strives to meet the needs of our learners and patients in creative ways while continuing to monitor our progress.

Emergency Medicine physicians have been shown to readily adapt to changing environments and new educational demands. While not comfortable or easy, EM educators have demonstrated commitment to developing the best educational environment for future emergency physicians. With changing financial support systems directly affecting GME,EM continues to be the innovators of medical education and still strives to balance the needs of our learners, the future needs of our patients and the financial burden of medical education.

### Conflict of Interest

The author declares that there are no conflicts of interest.
